# Prehospital PEEP for Acute Respiratory Distress: Protocol for a Scoping Review

**DOI:** 10.1111/aas.70314

**Published:** 2026-07-29

**Authors:** Daniel Wittrock, Joschka Martin Bauer, Daniel Hägi‐Pedersen, Helle Collatz Christensen, Søren Mikkelsen, Anne Craveiro Brøchner

**Affiliations:** ^1^ ZUPREME, Prehospital Centre Naestved Denmark; ^2^ Prehospital Research Unit Prehospital South—EMS Odense Denmark; ^3^ University of Southern Denmark Odense Denmark; ^4^ Research Centre of Anaesthesiology and Intensive Care Medicine (RCAI), Department of Anaesthesiology Central and West Zealand Hospital Næstved Denmark; ^5^ Department of Clinical Medicine University of Copenhagen Copenhagen Denmark; ^6^ Department of Anaesthesiology and Intensive Care Lillebaelt University Hospital Kolding Denmark

**Keywords:** acute respiratory distress, non‐invasive ventilation, positive end‐expiratory pressure, prehospital care, scoping review

## Abstract

**Background:**

Positive end‐expiratory pressure (PEEP) can improve oxygenation and reduce the work of breathing in patients with acute respiratory distress. In the prehospital setting, systems providing continuous positive airway pressure (CPAP), bi‐level positive airway pressure (BiPAP) and adjustable pressure‐limiting (APL) valves are used inconsistently, and the evidence base is heterogeneous across their use in patients with chronic obstructive pulmonary disease, asthma, pneumonia and cardiogenic pulmonary oedema.

**Purpose:**

This scoping review aims to map the existing evidence on prehospital use of PEEP‐based ventilation in adults with acute respiratory distress, including reported clinical outcomes and safety.

**Methods:**

This scoping review will be conducted in accordance with the JBI methodology for scoping reviews and reported in line with the PRISMA extension for Scoping Reviews (PRISMA‐ScR). We will systematically search MEDLINE, Embase, CINAHL, Cochrane CENTRAL and the Cochrane Database of Systematic Reviews (CDSR) with support from a research librarian. Empirical studies of adults (≥ 18 years) receiving PEEP‐based ventilation in the prehospital setting will be eligible, including randomised controlled trials, observational studies, case series and well‐described case reports. Two independent reviewers will screen, extract and map the data on study characteristics, interventions, comparators and outcomes, with disagreements resolved by a third reviewer. Methodological quality will be appraised at study level using the Jadad Scale for randomised trials and the Newcastle–Ottawa Scale for cohort and case–control studies.

**Anticipated Results:**

We expect to identify a limited but clinically relevant body of evidence reporting effects on oxygenation, dyspnoea severity, escalation to advanced airway management, adverse events and feasibility. The findings will be synthesised narratively and presented in descriptive and tabular form. We specifically anticipate sparse evidence for the prehospital use of APL systems.

**Conclusion:**

This scoping review will provide a comprehensive mapping of prehospital PEEP use in adults with acute respiratory distress, summarising its clinical effects, safety signals and feasibility, and identifying gaps to inform future studies.

**Abbreviations**
APL: adjustable pressure‐limiting
ARDS: acute respiratory distress syndrome
BiPAP: bi‐level positive airway pressure
CDSR: Cochrane Database of Systematic Reviews
CINAHL: cumulative index to nursing and allied health literature
COPD: chronic obstructive pulmonary disease
CPAP: continuous positive airway pressure
EMS: emergency medical services
EMT: emergency medical technician
GRADE: Grading of Recommendations Assessment, Development and Evaluation
MEDLINE: Medical Literature Analysis and Retrieval System Online
NIV: non‐invasive ventilation
PCC: population, concept and context
PEEP: positive end‐expiratory pressure
PEP: positive expiratory pressure
PRISMA‐P: Preferred Reporting Items for Systematic Review and Meta‐Analysis Protocols
PRISMA‐ScR: Preferred Reporting Items for Systematic Reviews and Meta‐Analyses extension for Scoping Reviews
PROSPERO: International Prospective Register of Systematic Reviews
SpO_2_
: peripheral oxygen saturation

## 1 Introduction

### 1.1 Rationale

Positive end‐expiratory pressure (PEEP) improves oxygenation and reduces the work of breathing in patients with acute respiratory distress [1, 2, 3]. However, evidence for the prehospital application of PEEP devices is limited and heterogeneous. Therefore, a scoping review is warranted to map and characterise the available evidence on clinical effectiveness, safety, feasibility and implementation associated with prehospital PEEP use in adult patients with dyspnoea due to chronic obstructive pulmonary disease, asthma, pneumonia or cardiogenic pulmonary oedema [4].

PEEP‐based treatment has been used for several decades in patients with acute respiratory distress syndrome (ARDS). Continuous positive airway pressure (CPAP) was introduced in the late 1970s [5] and has since been applied in a range of in‐hospital settings, including pulmonology wards, physiotherapy, intensive care units and operating theatres. Devices range from positive expiratory pressure (PEP) valves, operated by patients themselves, to adjustable pressure‐limiting (APL) valves and more advanced CPAP and bi‐level positive airway pressure (BiPAP) systems.

Early application of PEEP in acute respiratory distress may reduce the need for subsequent invasive ventilation, advanced respiratory support and admission to the intensive care unit in the receiving hospital [5, 6, 7]. It may also reduce the need for repeated β_2_‐agonist inhalations before arriving at the emergency department [4].

Given increasing operational interest in simple, widely available devices, APL valves warrant specific attention; however, we anticipate that the published prehospital evidence for APL is sparse. Given the heterogeneity of publicly accessible evidence, this scoping review is designed to map the extent, nature and key characteristics of this evidence.

### 1.2 Objectives

#### 1.2.1 Primary Objective

To map and summarise the literature on prehospital PEEP‐based ventilation in adults with acute respiratory distress, including reported clinical outcomes and safety.

The specific objectives include describing the study designs, populations, interventions (device types), comparators and outcomes; summarising the effects on oxygenation, dyspnoea and escalation to advanced airway management; identifying adverse events, feasibility issues and patient‐centred outcomes and highlighting knowledge gaps.

### 1.3 Research Questions

- Among adults with acute respiratory distress treated in the prehospital setting, what clinical outcomes are reported following PEEP‐based ventilation compared with standard care without PEEP (e.g., supplemental oxygen and β_2_‐agonist treatment)?
- What adverse effects, feasibility challenges and patient‐centred outcomes have been reported in relation to prehospital PEEP treatment?

Acute respiratory distress will be assessed using the scales and measures reported by the included studies, such as the Borg scale, numerical rating scales for dyspnoea and clinician‐rated dyspnoea scores.

## 2 Methods

The conduct of this scoping review will follow the JBI methodology for scoping reviews [8], and reporting will adhere to the PRISMA extension for Scoping Reviews checklist (PRISMA‐ScR) [9]. To minimise duplication and ensure that no recent or ongoing review overlapped with the scope of this review, we will search MEDLINE (via Ovid), Embase (via Ovid), CINAHL and Cochrane CENTRAL for registered trials and the Cochrane Database of Systematic Reviews (CDSR) for published and registered reviews via the Cochrane Library.

In accordance with the PROSPERO eligibility criteria, scoping reviews are not eligible for registration; therefore, this protocol will not be registered [10].

### 2.1 Eligibility Criteria

#### 2.1.1 Study Design

All empirical study designs will be eligible, including randomised controlled trials, quasi‐experimental studies, cohort and case–control studies, cross‐sectional studies and other observational designs. Case series and well‐described case reports will also be included.

#### 2.1.2 PCC‐Model

Since this review is categorised as a scoping review, we will formulate the review question according to the PCC‐model (population, concept and context) [9, 11, 12, 13]

- *Population*: Studies involving adult patients (≥ 18 years) with acute respiratory distress will be included. Underlying conditions may include, but are not limited to, COPD, asthma, pneumonia and cardiogenic pulmonary oedema. Studies restricted to in‐hospital patients and those in which prehospital and in‐hospital data cannot be separated will be excluded. Studies exclusively involving paediatric populations (< 18 years) will also be excluded. Studies including both adult and paediatric participants will be included if the adult data are reported separately or constitute a clear and interpretable majority.
- *Concept*: Patients treated with a PEEP‐intervention, without advanced or surgical airway in place.
- *Context*: in a prehospital setting.

We acknowledge that the composition of the prehospital workforce varies by country (e.g., physician‐staffed units, emergency nurses, paramedics, emergency medical technicians (EMTs) or personnel with limited healthcare training). Consequently, our search strategy will integrate both specific professional titles and broader terms related to the prehospital setting to enhance the likelihood of capturing all relevant studies, regardless of local role designations or terminology. We will not restrict inclusion based on professional labels used within individual articles in recognition of these established international variations.

In short, this scoping review will include studies regarding the application of PEEP in the prehospital setting using devices such as non‐invasive ventilation (NIV), BiPAP, CPAP, APL valves or other devices delivering PEEP or equivalent non‐invasive positive pressure ventilation.

### 2.2 Information Sources

We will search MEDLINE, Embase, CINAHL, the CDSR and Cochrane CENTRAL databases. Strategies will be adapted to each database with the assistance of a research librarian.

The reference lists of the included studies and relevant reviews will also be screened. Reviewers will not be blinded to the journal titles, authors or institutions.

No language restrictions will be applied; translation tools will be used as necessary (for languages other than Danish, English, German, French, Norwegian, Portuguese, Spanish and Swedish).

### 2.3 Search Strategy

A comprehensive search strategy will combine controlled vocabulary terms (as appropriate for each database) and free‐text terms for the three main concepts (profession, setting and intervention).

We will structure the search by combining relevant professions with prehospital and out‐of‐hospital settings. The search results will be combined with relevant interventions. Finally, we will exclude children and some specifics that do not fit the context and criteria of this scoping review.

A research librarian will assist with the search process for all databases. We will use Boolean operators to combine (‘OR’ or ‘AND’) the relevant search themes and to exclude (‘NOT’) specific words/subthemes. The search strategy, including the terms and free text, is provided in Supporting Information File S1.

### 2.4 Study Records

#### 2.4.1 Data Management

Covidence [14] will be used for reference management, deduplication, screening and data extraction.

#### 2.4.2 Selection Process

The study selection will be performed by two reviewers (D.W. and J.M.B.) who independently screen the titles, abstracts and full texts. Studies that appear to meet the inclusion criteria or studies with uncertain eligibility will be included in the full‐text screening. Disagreements will be resolved through discussion or by a third reviewer (A.C.B.). This process will be documented in a PRISMA flow diagram.

### 2.5 Quality Assessment

Whilst scoping reviews typically do not include outcome‐level risk of bias assessments (as outlined in the PRISMA‐ScR guidelines), we will perform a study‐level critical appraisal to provide a basic overview of methodological quality for the included randomised, case–control and cohort studies. This approach acknowledges the resource‐intensive nature of outcome‐level assessments whilst still allowing for a structured evaluation of study quality (Supporting Information File S2).

- *Randomised studies*: We will use the Jadad Scale (a study‐level tool) to assess randomisation, blinding and withdrawals/dropouts [15].
- *Case–control and cohort studies*: We will apply the Newcastle–Ottawa Scale (NOS) to evaluate selection, comparability and exposure/outcome measurement [16].

### 2.6 Data Items

Data extraction will be performed independently and in duplicate by two reviewers (D.W. and J.M.B.), with disagreements resolved by discussion or by a third reviewer (A.C.B.). The data items will include (as reported):

- *Demography*: Author(s), year of publication, country, setting, study design, funding and conflicts of interest.
- *Population (patients):* Sample size, age, sex at birth, underlying/presenting diagnosis/indication (e.g., COPD, asthma, pneumonia, cardiogenic pulmonary oedema), inclusion/exclusion criteria and additional indicators, such as illness severity (e.g., early warning score), Glasgow Coma Score, body mass index and comorbidities when available.
- *Concept*: Type of PEEP device (e.g., CPAP, APL valve), PEEP level(s), duration of treatment, provider type (EMTs, paramedic, emergency nurse, physician‐staffed unit).
- *Context*: The prehospital setting.

### 2.7 Data Synthesis

The primary approach will be descriptive mapping with tabular and narrative summaries grouped by diagnosis, device type, approximate PEEP level and EMS setting. Where two or more studies report sufficiently comparable populations, interventions and outcomes, their results will be summarised side by side in tabular form and described narratively, without statistical pooling, but we will conduct a descriptive synthesis of quality evidence [17].

## 3 Discussion

In this scoping review, we aim to assess and map the current evidence on the use of PEEP in the prehospital setting in adult patients with acute respiratory distress. This review provides an overview of the existing knowledge, describes how PEEP is currently used and evaluated in prehospital care and identifies unanswered research questions and evidence gaps. This, in turn, is intended to inform the design and prioritisation of future prehospital trials and implementation studies on PEEP‐based treatment.

The strengths of this review include a PCC‐based systematic search strategy, clearly defined eligibility criteria and a transparent, reproducible approach to charting and mapping the available evidence.

The pre‐specification of the methods in this protocol enhances its transparency and reproducibility.

This scoping review has several limitations. We anticipate a relatively small and heterogeneous evidence base, which may preclude a robust quantitative synthesis and limit the ability to draw firm conclusions regarding its effectiveness. Methodological quality will be appraised at study level rather than at outcome level. In keeping with scoping‐review methodology, PRISMA‐ScR does not require a formal risk‐of‐bias assessment; our use of the Jadad Scale and the Newcastle–Ottawa Scale is therefore intended to provide a basic overview of study quality rather than to weight, downgrade or exclude studies.

This study‐level appraisal may be neither feasible nor meaningful for all designs, particularly case series and well‐described case reports, for which quality will be described narratively. Any GRADE assessment of certainty is contingent on a feasible quantitative synthesis; given the anticipated sparse and heterogeneous evidence, certainty in the body of evidence may instead be appraised only qualitatively.

Furthermore, our intentionally broad search strategy may have resulted in substantial methodological and clinical heterogeneity between studies. However, this breadth is necessary to ensure comprehensive mapping of the literature. Despite these limitations, this review provides an important foundation by collating and organising what is currently known about prehospital PEEP use in patients with acute respiratory distress.

## Author Contributions

Conceptualisation: D.W. Methodology: D.W., J.M.B., A.C.B. Validation: A.C.B., S.M., H.C.C., D.H.‐P. Formal analysis: D.W., J.M.B. Investigation: D.W., J.M.B. Data curation: D.W., J.M.B. Writing – original draft: D.W. Writing – review and editing: J.M.B., S.M., H.C.C., D.H.‐P., A.C.B. Supervision: A.C.B. Guarantor: D.W.

## Funding

This review will be conducted without any dedicated external funding. Institutional support is provided by the Prehospital Research Unit, Region of Southern Denmark and the Prehospital Center, Region Zealand, Denmark.

## Disclosure

Any substantial amendments to this protocol (e.g., changes to eligibility criteria, outcomes or analysis plan) will be documented with dates and rationales and reported in the final manuscript (and in any publicly accessible protocol repository, if used).

## Ethics Statement

This review uses previously published data only and does not involve human participants or identifiable data; therefore, formal ethical approval and individual consent are not required for this study.

## Conflicts of Interest

The authors declare no conflicts of interest.

## Supporting information


**Supporting Information: File S1.** Search scheme.

**Supporting Information: File S2.** Preferred Reporting Items for Systematic reviews and Meta‐Analyses extension for Scoping Reviews (PRISMA‐ScR) Checklist.

## Data Availability

The data that support the findings of this study are available from the corresponding author upon reasonable request.
