# Supplementary material for: Prehospital PEEP for Acute Respiratory Distress: Protocol for a Scoping Review
Source: Acta Anaesthesiol Scand. 2026 Jul 29;70(8):e70314. doi: 10.1111/aas.70314 (PMC13420747; doi:10.1111/aas.70314)
Supplement: Supplementary file 1 — Supporting Information: File S1. Search scheme. [file AAS-70-0-s002.docx]

# Supplementary 1 – Search scheme

This is an overview, showing our search strategy for MEDLINE, EMBASE, CINAHL, Cochrane CENTRAL and Cochrane Database of Systematic Reviews (CDSR).

**Search strategy**

*(((‘Profession’ OR ‘Setting’) AND ‘Intervention’) NOT ‘Exclusions’)*

|  | **MEDLINE** | |
| --- | --- | --- |
|  | *MeSH terms* | *Free text (title, abstract, keyword)* |
| Profession | Paramedics/ OR  Emergency Medical Technicians/ OR  Emergency Responders/ OR  Rescue Work/ | (paramedic* OR paramedical OR "ambulance clinician*" OR "ambulance staff" OR "ambulance personnel" OR "ambulance crew*"  OR "emergency medical technician*"  OR emt OR emr OR "pre-hospital nurse*" OR "prehospital nurse*" OR  "flight paramedic*" OR "flight nurse*" OR "emergency care practitioner*" OR "emergency service personnel" OR "emergency nurse" OR "prehospital clinician*" OR "emergency care assistant*" OR  "emergency medical service personnel").ti,ab,kf. |
| Setting | *Emergency Medical Services/ or Ambulances/ or Air Ambulances/ or *Transportation of Patients/ or Paramedics/ or Emergency Medical Technicians/ or Emergency Responders/ | ("emergency medical service" or "emergency medical services" or ems or "emergency medical system*" or ambulance* or "ambulance service*" or prehospital or pre-hospital or "prehospital care" or "pre-hospital care" or "prehospital emergency care" or "out of hospital" or out-of-hospital or "out-of-hospital care" or "first responder*" or "emergency responder*" or on-scene or en-route or in-transit or "air ambulance*" or "air medical" or aeromedical or hems or "helicopter emergency medical service*" or "medical evacuation" or medevac or "aeromedical evacuation" or "patient transport*" or "transportation of patients" or "patient transportation" or "interfacility transport*" or "critical care transport*" or "medical transport*" or micu or "mobile intensive care unit*").ti,ab,kf. |
| Intervention | Noninvasive Ventilation/ OR Positive-Pressure Respiration/ OR Continuous Positive Airway Pressure/ OR Intermittent Positive-Pressure Breathing/ OR Intermittent Positive-Pressure Ventilation/ | (peep or "positive end-expiratory pressure" or "positive end expiratory pressure" or "end-expiratory pressure" or "end expiratory pressure" or epap or "positive expiratory pressure" or "expiratory positive airway pressure" or "peep valve*" or "positive-pressure respiration*" or "positive pressure ventilation*" or "positive end expiratory pressure*" or ((noninvasive or "non invasive" or non-invasive) adj2 (ventilat* or "respiratory support" or "ventilatory support" or "respiratory assistance" or "breathing support" or "positive pressure")) or niv or nippv or nppv or "continuous positive airway pressure" or "constant positive airway pressure" or cpap or ncpap or "nasal cpap" or "airway pressure release ventilation" or aprv or "intermittent positive pressure ventilation" or ippv or "intermittent positive pressure breathing" or ippb or "bilevel positive airway pressure" or bipap or bpap or "bi-level* positive airway pressure" or "biphasic positive airway pressure" or "positive pressure ventilation" or "positive-pressure ventilation" or "adjustable pressure limiting valve*" or "adjustable pressure-limiting valve*" or "pressure limiting valve*" or "pressure-limiting valve*" or "adjustable pressure limit* valve*" or "apl valve*" or apl).ti,ab,kw,kf. or ("airway management" or "mechanical ventilation").ti. |
| Exclusions | NOT (exp animals/ or exp animal experimentation/ or exp animal experiment/ or exp models animal/ or exp vertebrate/ or exp vertebrates/)  NOT  (exp humans/ or exp human experimentation/ or exp human experiment/) | NOT ((children or child or boy or boys or girl or girls or teen or teenager* or neonate* or infant or infants or baby or babies or toddler* or preschool* or adolescent* or pediatric* or paediatric* or youth*).ti. or exp juvenile/) not (exp adult/ or (adult* or men or women or patient* or elderly or senior* or geriatric* or old or aging or ageing or aged or sexagenarian* or septuagenarian* or octogenarian* or nonagenarian* or centenarian*).ti.)  (exp animals/ or exp animal experimentation/ or exp animal experiment/ or exp models animal/ or exp vertebrate/ or exp vertebrates/ |

|  | **Embase** | |
| --- | --- | --- |
|  | Emtree | *Free text (title, abstract, keyword)* |
| Profession | paramedical personnel/ OR paramedical profession/ OR rescue personnel/ | (paramedic* or paramedical or "ambulance clinician*" or "ambulance staff" or "ambulance personnel" or "ambulance crew*" or "emergency medical technician*" or emt or emr or "pre-hospital nurse*" or "prehospital nurse*" or "flight paramedic*" or "flight nurse*" or "emergency care practitioner*" or "emergency service personnel" or "emergency nurse" or "prehospital clinician*" or "emergency care assistant*" or "emergency medical service personnel").ti,ab,kf. |
| Setting | *emergency health service/ or air medical transport/ or ambulance/ or helicopter emergency medical service/ or *patient transport/ | ("emergency medical service*" or "emergency medical services" or ems or "emergency medical system*" or ambulance* or "ambulance service*" or prehospital or pre-hospital or "prehospital care" or "pre-hospital care" or "prehospital emergency care" or "out of hospital" or out-of-hospital or "out-of-hospital care" or "first responder*" or "emergency responder*" or on-scene or en-route or in-transit or ("air ambulance*" or "air medical" or aeromedical or hems or "helicopter emergency medical service*" or "medical evacuation" or medevac or "aeromedical evacuation")).mp. or ("patient transport*" or "transportation of patients" or "patient transportation" or "interfacility transport*" or "critical care transport*" or "medical transport*" or micu or "mobile intensive care unit*").ti,ab,kf. |
| Intervention | positive end expiratory pressure ventilation/ or bilevel positive airway pressure/ or automatic positive airway pressure/ or airway pressure release ventilation/ or noninvasive ventilation/ or positive pressure ventilation/ or *artificial ventilation/ or continuous positive airway pressure/ or intermittent positive pressure ventilation/ or noninvasive positive pressure ventilation/ or *respiratory control/ | (peep or "positive end-expiratory pressure" or "positive end expiratory pressure" or "end-expiratory pressure" or "end expiratory pressure" or epap or "positive expiratory pressure" or "expiratory positive airway pressure" or "peep valve*" or "positive-pressure respiration*" or "positive pressure ventilation*" or "positive end expiratory pressure*" or ((noninvasive or "non invasive" or non-invasive) adj2 (ventilat* or "respiratory support" or "ventilatory support" or "respiratory assistance" or "breathing support" or "positive pressure")) or niv or nippv or nppv or "continuous positive airway pressure" or "constant positive airway pressure" or cpap or ncpap or "nasal cpap" or "airway pressure release ventilation" or aprv or "intermittent positive pressure ventilation" or ippv or "intermittent positive pressure breathing" or ippb or "bilevel positive airway pressure" or bipap or bpap or "bi-level* positive airway pressure" or "biphasic positive airway pressure" or "positive pressure ventilation" or "positive-pressure ventilation" or "adjustable pressure limiting valve*" or "adjustable pressure-limiting valve*" or "pressure limiting valve*" or "pressure-limiting valve*" or "adjustable pressure limit* valve*" or "apl valve*" or apl).ti,ab,kw,kf. or ("airway management" or "mechanical ventilation").ti. |
| Exclusions |  | not (infant* or child* or neonat* or (postoperative or "acute myeloid leukaemia" or exercise or training or cat* or dog* or rat* or mouse or mice)).ti,ab,kw,kf. |

|  | **CINAHL** | |
| --- | --- | --- |
|  | *CINAHL Headings* | *Free text (title, abstract)* |
| Profession | (MH Paramedics) OR (MH Emergency Medical Technicians) OR (MH Allied Health Personnel) OR (MH Flight Nurses) OR (MH Medical Flight Specialists) OR (MH Flight Nursing) | (XB paramedic* OR XB paramedical OR XB "ambulance clinician*" OR XB "ambulance staff" OR XB "ambulance personnel" OR XB "ambulance crew*" OR XB "emergency medical technician*" OR XB emt OR XB emr OR XB "pre-hospital nurse*" OR XB "prehospital nurse*" OR XB "flight paramedic*" OR XB "flight nurse*" OR XB "emergency care practitioner*" OR XB "emergency service personnel" OR XB "emergency nurse" OR XB "prehospital clinician*" OR XB "emergency care assistant*" OR XB "emergency medical service personnel")) |
| Setting | (MH Emergency Medical Services+) OR (MH Ambulances) OR (MH Transportation of Patients+) OR (MH Prehospital Care) | ("emergency medical service*" OR "emergency medical services" OR ems OR "emergency medical system*" OR ambulance* OR "ambulance service*" OR prehospital OR pre-hospital OR "prehospital care" OR "pre-hospital care" OR "prehospital emergency care" OR "out of hospital" OR out-of-hospital OR "out-of-hospital care" OR "first responder*" OR "emergency responder*" OR on-scene OR en-route OR in-transit OR "air ambulance*" OR "air medical" OR aeromedical OR hems OR "helicopter emergency medical service*" OR "medical evacuation" OR medevac OR "aeromedical evacuation" OR XB "patient transport*" OR XB "transportation of patients" OR XB "patient transportation" OR XB "interfacility transport*" OR XB "critical care transport*" OR XB "medical transport*" OR XB micu OR XB "mobile intensive care unit*"))) |
| Intervention | (MH Positive Pressure Ventilation+) OR (MH Noninvasive Ventilation+) OR (MH Respiration, Artificial+) OR (MH Continuous Positive Airway Pressure+) OR (MH Intermittent Positive Pressure Ventilation+) OR (MH Intermittent Positive Pressure Breathing+) | (peep OR "positive end-expiratory pressure" OR "positive end expiratory pressure" OR "end-expiratory pressure" OR "end expiratory pressure" OR epap OR "positive expiratory pressure" OR "expiratory positive airway pressure" OR "peep valve*" OR "positive-pressure respiration*" OR "positive pressure ventilation*" OR "positive end expiratory pressure*" OR ((noninvasive OR "non invasive" OR non-invasive) N2 (ventilat* OR "respiratory support" OR "ventilatory support" OR "respiratory assistance" OR "breathing support" OR "positive pressure")) OR niv OR nippv OR nppv OR "continuous positive airway pressure" OR "constant positive airway pressure" OR cpap OR ncpap OR "nasal cpap" OR "airway pressure release ventilation" OR aprv OR "intermittent positive pressure ventilation" OR ippv OR "intermittent positive pressure breathing" OR ippb OR "bilevel positive airway pressure" OR bipap OR bpap OR "bi-level* positive airway pressure" OR "biphasic positive airway pressure" OR "positive pressure ventilation" OR "positive-pressure ventilation" OR XB "adjustable pressure limiting valve*" OR XB "adjustable pressure-limiting valve*" OR XB "pressure limiting valve*" OR XB "pressure-limiting valve*" OR XB "adjustable pressure limit* valve*" OR XB "apl valve*" OR XB apl))) |
| Exclusions |  | ((TI (children OR child OR boy OR boys OR girl OR girls OR teen OR teenager* OR neonate* OR infant OR infants OR baby OR babies OR toddler* OR preschool* OR adolescent* OR pediatric* OR paediatric* OR youth*) or MH "Child+" OR MH "Adolescence+" OR MH "Infant+") not (MH "Adult+" or (TI adult* or men or women or patient* or elderly or senior* or geriatric* or old or aging or ageing or aged or sexagenarian* or septuagenarian* or octogenarian* or nonagenarian* or centenarian*) |

|  | **Cochrane Library & Cochrane CENTRAL** | |
| --- | --- | --- |
|  | *MeSH* | *Free text (title, abstract, keyword)* |
| Profession | MeSH descriptor: [Paramedics] explode all trees  MeSH descriptor: [Emergency Medical Technicians] explode all trees  MeSH descriptor: [Emergency Responders] explode all trees  MeSH descriptor: [Rescue Work] explode all trees | (( paramedic*:ti,ab,kw OR paramedical:ti,ab,kw OR ("ambulance" NEAR/1 clinician*):ti,ab,kw OR "ambulance staff":ti,ab,kw OR "ambulance personnel":ti,ab,kw OR ("ambulance" NEAR/1 crew*):ti,ab,kw OR ("emergency medical" NEAR/1 technician*):ti,ab,kw OR emt:ti,ab,kw OR emr:ti,ab,kw OR ("pre-hospital" NEAR/1 nurse*):ti,ab,kw OR ("prehospital" NEAR/1 nurse*):ti,ab,kw OR ("flight" NEAR/1 paramedic*):ti,ab,kw OR ("flight" NEAR/1 nurse*):ti,ab,kw OR ("emergency care" NEAR/1 practitioner*):ti,ab,kw OR "emergency service personnel":ti,ab,kw OR "emergency nurse":ti,ab,kw OR ("prehospital" NEAR/1 clinician*):ti,ab,kw OR ("emergency care" NEAR/1 assistant*):ti,ab,kw OR "emergency medical service personnel":ti,ab,kw )):ti,ab,kw |
| Setting | MeSH descriptor: [Emergency Medical Services] explode all trees  MeSH descriptor: [Ambulances] explode all trees  MeSH descriptor: [Air Ambulances] explode all trees  MeSH descriptor: [Transportation of Patients] explode all trees  MeSH descriptor: [Paramedics] explode all trees  MeSH descriptor: [Emergency Medical Technicians] explode all trees  MeSH descriptor: [Emergency Responders] explode all trees | (( (("emergency medical" NEAR/1 service*) OR "emergency medical services" OR ems OR ("emergency medical" NEAR/1 system*) OR ambulance* OR ("ambulance" NEAR/1 service*) OR prehospital OR pre-hospital OR "prehospital care" OR "pre-hospital care" OR "prehospital emergency care" OR "out of hospital" OR out-of-hospital OR "out-of-hospital care" OR ("first" NEAR/1 responder*) OR ("emergency" NEAR/1 responder*) OR on-scene OR en-route OR in-transit ) OR ( ("air" NEAR/1 ambulance*) OR "air medical" OR aeromedical OR hems OR ("helicopter emergency medical" NEAR/1 service*) OR "medical evacuation" OR medevac OR "aeromedical evacuation" ) OR ( ("patient" NEAR/1 transport*):ti,ab,kw OR "transportation of patients":ti,ab,kw OR "patient transportation":ti,ab,kw OR ("interfacility" NEAR/1 transport*):ti,ab,kw OR ("critical care" NEAR/1 transport*):ti,ab,kw OR ("medical" NEAR/1 transport*):ti,ab,kw OR micu:ti,ab,kw OR ("mobile intensive care" NEAR/1 unit*):ti,ab,kw ) )):ti,ab,kw |
| Intervention | MeSH descriptor: [Noninvasive Ventilation] explode all trees  MeSH descriptor: [Positive-Pressure Respiration] explode all trees  MeSH descriptor: [Respiration, Artificial] explode all trees  MeSH descriptor: [Continuous Positive Airway Pressure] explode all trees  MeSH descriptor: [Intermittent Positive-Pressure Ventilation] explode all trees  MeSH descriptor: [Intermittent Positive-Pressure Breathing] explode all trees  MeSH descriptor: [Biphasic Continuous Positive Airway Pressure] explode all trees | (( peep OR "positive end-expiratory pressure" OR "positive end expiratory pressure" OR "end-expiratory pressure" OR "end expiratory pressure" OR epap OR "positive expiratory pressure" OR "expiratory positive airway pressure" OR ("peep" NEAR/1 valve*) OR ("positive-pressure" NEAR/1 respiration*) OR ("positive pressure" NEAR/1 ventilation*) OR ("positive end expiratory" NEAR/1 pressure*) ) OR ( ((noninvasive OR "non invasive" OR non-invasive) NEAR/2 (ventilat* OR "respiratory support" OR "ventilatory support" OR "respiratory assistance" OR "breathing support" OR "positive pressure")) OR niv OR nippv OR nppv ) OR ( "continuous positive airway pressure" OR "constant positive airway pressure" OR cpap OR ncpap OR "nasal cpap" OR "airway pressure release ventilation" OR aprv OR "intermittent positive pressure ventilation" OR ippv OR "intermittent positive pressure breathing" OR ippb OR "bilevel positive airway pressure" OR bipap OR bpap OR (bi-level* NEAR/1 "positive airway pressure") OR "biphasic positive airway pressure" OR "positive pressure ventilation" OR "positive-pressure ventilation" ) OR ( ("adjustable pressure limiting" NEAR/1 valve*):ti,ab,kw OR ("adjustable pressure-limiting" NEAR/1 valve*):ti,ab,kw OR ("pressure limiting" NEAR/1 valve*):ti,ab,kw OR ("pressure-limiting" NEAR/1 valve*):ti,ab,kw OR ("adjustable pressure" NEAR/1 limit* NEAR/1 valve*):ti,ab,kw OR ("apl" NEAR/1 valve*):ti,ab,kw OR apl:ti,ab,kw )):ti,ab,kw |
| Exclusions |  | NOT ((children:ti OR child:ti OR boy:ti OR boys:ti OR girl:ti OR girls:ti OR teen:ti OR teenager*:ti OR neonate*:ti OR infant:ti OR infants:ti OR baby:ti OR babies:ti OR toddler*:ti OR preschool*:ti OR adolescent*:ti OR pediatric*:ti OR paediatric*:ti OR youth*:ti) OR [mh child] OR [mh adolescent] OR [mh infant]) NOT ([mh adult] OR (adult*:ti OR men:ti OR women:ti OR patient*:ti OR elderly:ti OR senior*:ti OR geriatric*:ti OR old:ti OR aging:ti OR ageing:ti OR aged:ti OR sexagenarian*:ti OR septuagenarian*:ti OR octogenarian*:ti OR nonagenarian*:ti OR centenarian*:ti)) |
